# A Medical Causerie

**Published:** 1915-11-27

**Authors:** 


### A MEDICAL CAUSERIE.

The agitation relative to the enlistment of the junior medical student seems to have nearly, if not quite, subsided. After the War Office, the Director of Recruiting, and the Director-General of the R.A.M.C. had given a unanimous and considered decision, the majority of the profession doubtless considered the question settled, or they agreed, at all events, that in the circumstances no advantage was to be gained by further public discussion. Yet a few independent spirits have attempted to assert an individual judgment and in defiance of the authorities. On its merits the question is manifestly one on which much may be said, both in one direction and the other. But when the most prominent medical opinion had been duly considered by the Army authorities prior to the final decision, continued opposition is not easily justified and is a very doubtful kindness to the anxious student. The case for dissent, moreover, has not always been stated with scientific restraint. Thus one of the critics has drawn a truly harrowing picture of the consequences which may result from the recent decision. "Epidemics," we are reminded, "not infrequently follow war"; and on this possibility "the present proposals . . . will allow our mothers and children to suffer, and even perish, and will leave disease an unchecked career throughout the land." Yet according to the same authority the enlistment of junior medical students will, as a positive gain, mean "at the outside . . . a few hundred" added to Lord Derby's recruits. At the very earliest, the students who are asked to join the Colours could become qualified practitioners in two, three, and four years' time, and it is difficult to see how their enlistment can mean that "bad health will lead to a restricted output of munitions, to a diminished efficiency in those who are joining the fighting-line, to an unnecessary loss of life, to much avoidable suffering, to the increase of our National Debt, and, above all, to the postponement of victory or defeat." Admittedly, prior to the decision of the Army authorities there was something to be said for leaving medical students outside the recruiting scheme, but it may be doubted whether advocacy such as the above could be helpful in any circumstances. It has not received much support from medical teachers generally, who, whatever their individual opinions, have recognised the patriotic claim for accepting under present conditions the verdict of the powers that be. The plea for postponing the date on which individual students who are about to present themselves for a professional examination should be called to the ranks can be dealt with by the local committees, and will, no doubt, be treated with much respect; it does not affect the general decision, which ought now to be accepted as final. Perhaps the necessity of doing as we are told must be recognised as one of the "minor horrors" of the war.

The public, it appears, is threatened not only with a short supply of doctors, but also with a similar failure of qualified dentists. When the Dental Register was first set up—that is thirty-six years ago—the number entitled to registration, including those in practice prior to the passing of the Act, was 5,289, and in 1915 the total had only increased to 5,426. In considering these numbers it must be remembered that the original registration was necessarily conducted in a very catholic spirit, so that many persons whose claim to "qualification" was a somewhat slender one received statutory recognition. Now, every person who wishes to be registered as a qualified dentist must conform to the compulsory curriculum and pass the prescribed examinations. Making allowances for this want of correspondence between the two groups of figures just quoted, there still seems good reason to believe that the dental profession is not proving a very attractive one to adventurous youth. The various licensing bodies have been consulted and have submitted reports to the Dental Education Committee of the General Medical Council. While some blame is placed on the stringency and length of the curriculum and on the severity of the examinations, there is universal agreement that the principal cause of any deficiency in the supply of students is due to the inefficient protection afforded by the law to those who go to the trouble and expense of securing "registration." The public, it seems, do not appreciate the difference between the "qualified" man and the unqualified practitioner; and the latter may cultivate illuminations, window-cases, and other forms of advertisement which to the genuine Simon Pure are forbidden. Thus business men, having become acquainted with this state of matters, have come to the conclusion that qualification is worthless, or even a hindrance, and do not send their sons to the dental school. Indeed, according to one authority, even members of the profession prefer to have their sons educated by unqualified dentists as forming a more probable avenue to success. As an instance of the ease with which at least the spirit of the law is evaded, it seems that while none but a duly qualified person may announce himself as "Dental surgeon," it is open to any person to put after his name on a brass plate the term "Dental surgery." Even a judicial member of the public might be deceived by such a subterfuge, while the victim of a raging toothache can hardly stop to balance phrases. The War Office seems to favour the unqualified "dentists," who are, we regret to find, largely employed for Army purposes. Where does the soldier come in?

The transference of the House of the General Medical Council to Hallam Street may be said to emphasise the medical character of the Cavendish Square district. The street runs parallel to Harley Street, and the two are separated by Portland Place. At present the surroundings of the new House are not very impressive, but it is understood that the present leases are nearly at an end and that much more dignified buildings will take their place.

